# Vascular graft infection with duodenal fistulization 10 years after hybrid endovascular aortic repair with renovisceral debranching: a case report

**DOI:** 10.1186/s44215-023-00087-6

**Published:** 2023-08-18

**Authors:** Hiroaki Osada, Kazuhiro Yamazaki, Takahide Takeda, Kenji Minatoya

**Affiliations:** Department of Cardiovascular Surgery, Graduate School of Medicine, Kyoto University, 54 Shogoin-Kawaharacho, Sakyo-Ku, Kyoto, 606-8507 Japan

**Keywords:** Vascular grafting, Endovascular procedures, Fistula, Infection control

## Abstract

**Background:**

A secondary aortoenteric fistula after an aortic graft replacement or endovascular aortic repair is a rare but devastating complication that leads to extremely high morbidity and mortality. Because reports of vascular graft infection with enteric fistulization complicating in a hybrid aortic repair with renovisceral debranching are limited, the management method such as the extent of removal of the infected graft, reconstruction procedures, and the ideal type of graft are still debatable.

**Case presentation:**

We report a successful case of a 73-year-old man presenting a vascular graft infection with duodenal fistulization, 10 years after hybrid endovascular aortic repair with renovisceral debranching for a 60-mm diameter of supra-renal abdominal aortic aneurysm. The patient had a history of polymyalgia rheumatica on oral prednisolone, perigraft seroma, deep vein thrombosis, and an allergy to rifampicin. The patient eventually recovered after partial removal of the grafts, in situ reconstruction using Fusion Bioline vascular prosthesis, primary duodenal repair, application of omental flap, and antibiotics without any evidence of re-infection after 1.5 years.

**Conclusions:**

Although hybrid endovascular aortic repair is considered advantageous, especially for the elderly and high-risk patients, due to the avoidance of extracorporeal circulation and thoracotomy; once a devastating complication happens, an optimal treatment method should be considered for patients with several comorbidities. Although our procedures provided favorable results, careful monitoring to avoid re-infection is mandatory.

## Background

A secondary aortoenteric fistula is a complication present in 2% of open surgical abdominal aortic repairs [1] and 0.8% of endovascular aortic repairs (EVARs) [2], eventually leading to a high morbidity and mortality [3, 4]. Although relevant guidelines have outlined the clinical management of these devastating conditions [5], reports of vascular graft infection with enteric fistulization complicating hybrid aortic repairs with renovisceral debranching are limited [6]. In addition, the management method such as the extent of removal of the infected graft, reconstruction methods, and the types of grafts are still debatable. We report a successful case of a 73-year-old man with a vascular graft infection with duodenal fistulization, 10 years after a hybrid endovascular aortic repair with renovisceral debranching for a supra-renal abdominal aortic aneurysm.

## Case presentation

**Fig. 1:**
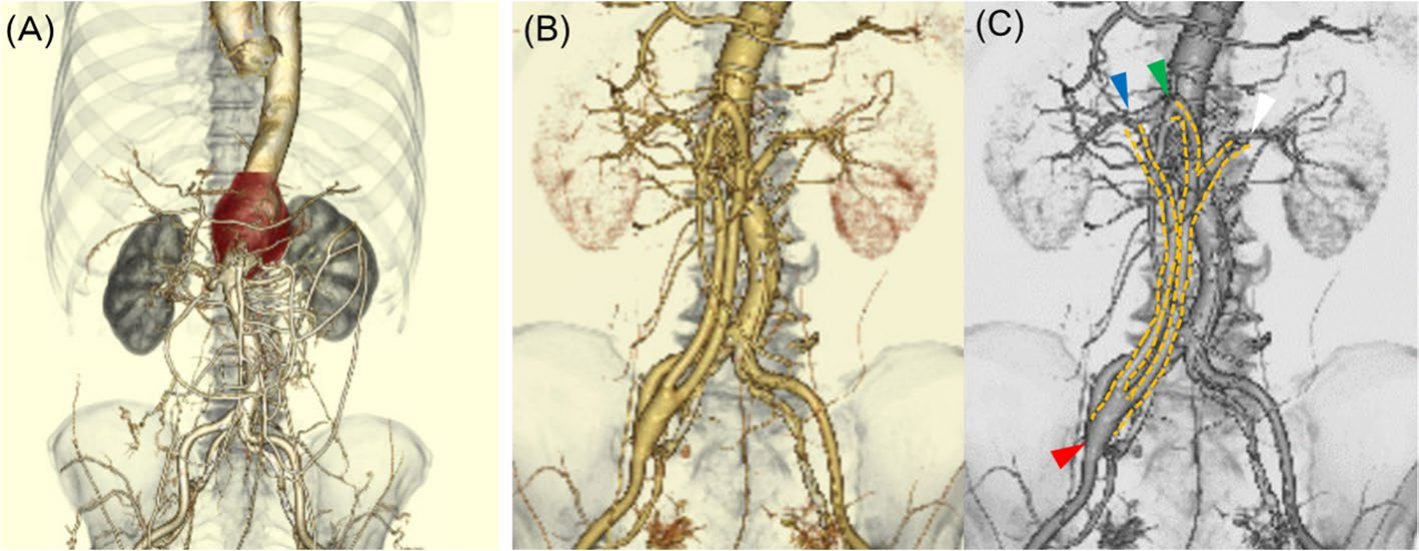
**A** Preoperative 3-dimensional CT angiography before the initial surgery 10 years ago of supra-renal abdominal aortic aneurysm which was 60 mm in diameter. The celiac artery originated from the aneurysm and occluded. **B**, **C** After initial surgery (renovisceral debranching and aortic stent-graft placement) in 2 different colors, the proximal side of 12 × 6 mm bifurcated GORE-TEX Vascular Graft (W. L. Gore & Associates, Inc. Flagstaff, AZ, USA) (yellow dotted line) was anastomosed to the right common iliac artery (red arrowhead), distal end of bifurcated graft was anastomosed to the right (blue arrowhead), and left renal artery (white arrowhead), respectively. A straight graft (green arrowhead) was placed between the superior mesenteric artery and the bifurcated graft toward the left renal artery. Six days after vascular graft placement, we placed a 180-mm length of INOUE STENTGRAFT between descending aorta and the abdominal aorta which is the level of Th11-L4. The proximal diameter of the stent graft was 30 mm, and the distal diameter was 20 mm

A 73-year-old man with a history of hypertension, polymyalgia rheumatica (PMR) on oral prednisolone for more than 10 years, who was referred to our department with a 2-month history of high fever of unknown origin. The other complications included a perigraft seroma, history of deep vein thrombosis, and an allergy to rifampicin. He had undergone a hybrid endovascular aortic repair with renovisceral debranching for a 60-mm-diameter supra-renal abdominal aortic aneurysm 10 years ago (Fig. 1A–C).

**Fig. 2:**
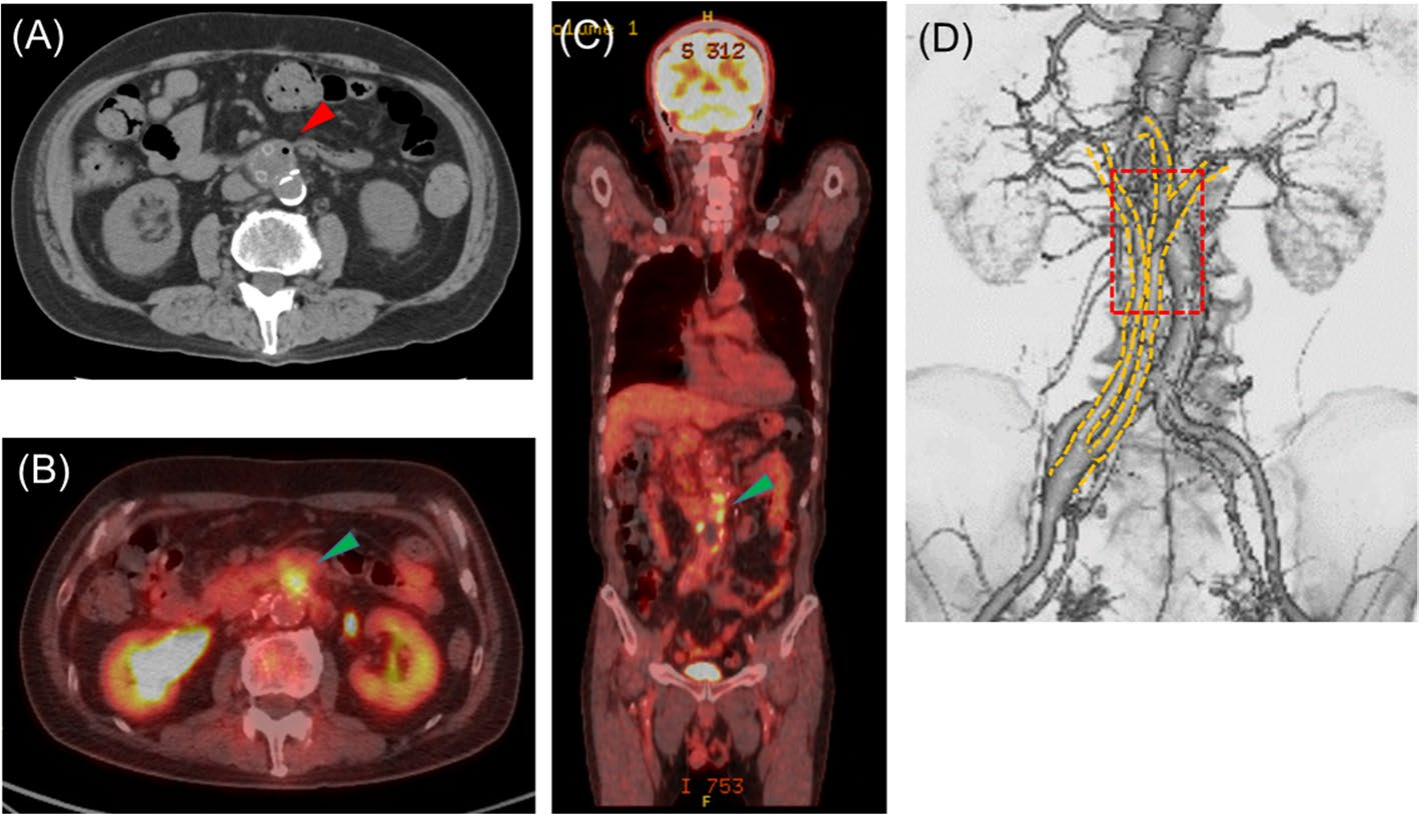
**A** Abdominal CT before surgery revealed encapsulated fluid and air collection around the grafts (red arrowhead). **B**, **C** Representative axial and coronal view of FDG-PET images which revealed focal uptake, SUVmax (maximum standardized uptake value) was 4.7–7.7 (green arrowhead). **D** We speculated infected area spread in the red-dotted line area by FDG-PET images

Laboratory data upon admission revealed an elevated white blood cell count (WBC) level of 12,770 /μL, C-reactive protein (CRP) level of 16.6 mg/dL, and procalcitonin (PCT) level of > 100 ng/mL. Initially, an acute exacerbation of his PMR and bacteremia associated with urinary tract infection (*Enterococcus faecalis* and *Streptococcus anginosus* were isolated from blood culture) were considered, and Ampicillin/Sulbactam and Tazobactam/Piperacillin were administered for more than 6 weeks. However, the fever did not resolve (repeated fever spikes in the 39 °C range); therefore, the patient was referred to our department because of strong suspicion of vascular graft infection. Abdominal computed tomography (CT) showed encapsulated fluid and air collection around the grafts (Fig. 2A). ^18^F-fluorodeoxyglucose positron emission tomography (FDG-PET) showed focal uptake of FDG localized in the same area (Fig. 2B–D). Additionally, upper gastrointestinal endoscopy revealed edema and a healed ulcer lesion in the third portion of the duodenum.

**Fig. 3:**
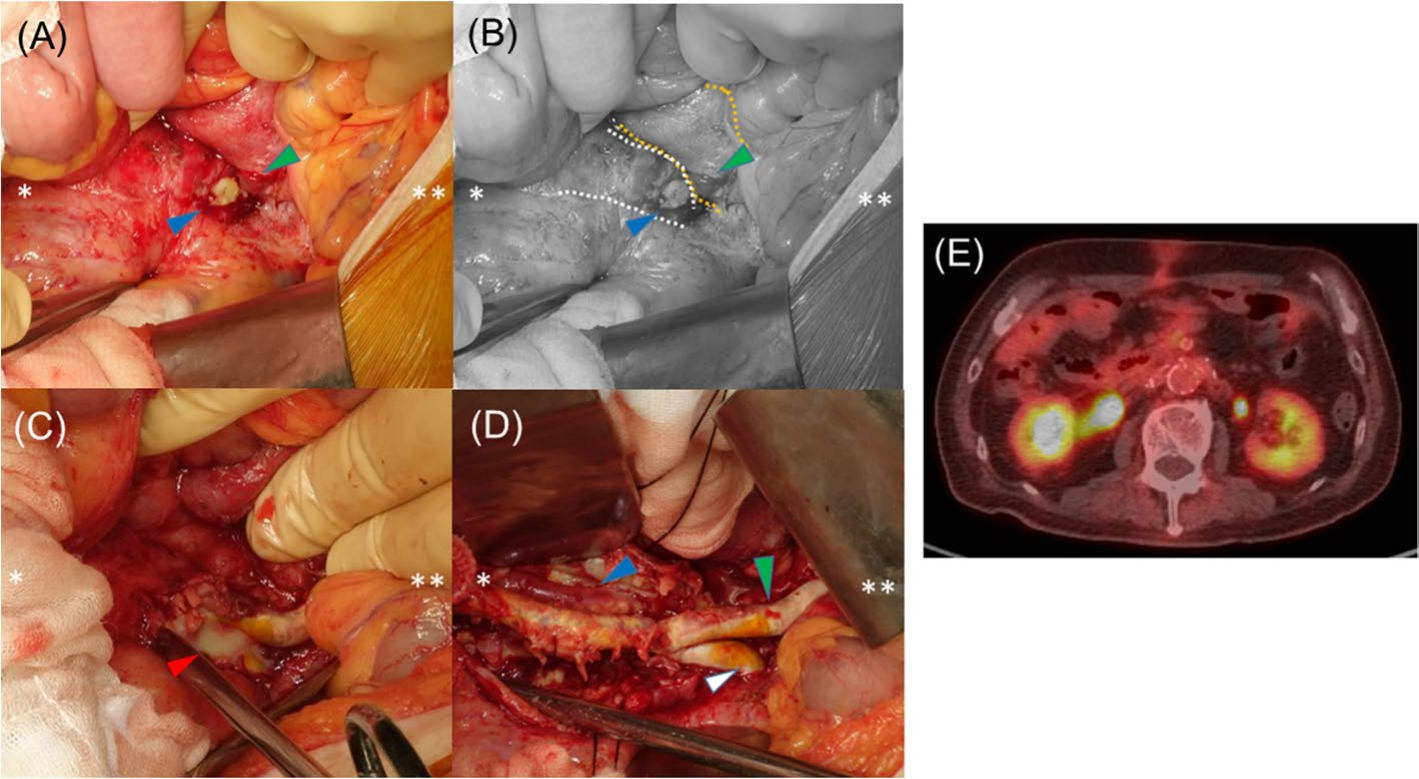
Intraoperative findings. **Cranial side. *Caudal side. **A**, **B** A duodenal fistulization (green arrowhead) and fistula connected to capsulated fluid collection area (blue arrowhead and white-dotted line). The horizontal part of the duodenum is represented by a yellow-dotted line. **C** Opened capsulated area with purulent discharge (red arrowhead) which was 7 cm in diameter. **D** After the debridement and irrigation of the same area before in situ reconstruction. Vascular grafts toward the right renal artery (blue arrowhead), left renal artery (white arrowhead), and superior mesenteric artery (green arrowhead). We replaced the vascular grafts in this visible part and the rest of the grafts which seemed to be highly adhesive were preserved. **E** Representative postoperative FDG-PET image which revealed no focal uptake

We perform surgical treatment for the vascular graft infection with duodenal fistulization. Through a median laparotomy, a 10-mm-diameter fistula between the encapsulated area and the horizontal part of the duodenum was identified (Fig. 3A, B), without bleeding nor vascular graft injury; however, massive purulent discharge around the grafts was observed (Fig. 3C). We employed primary suture repair for the duodenal fistula (Albert-Lembert procedure using 4–0 PDS). Tissues involved in the infection were debrided as much as possible and irrigated (Fig. 3D). Since the infection seemed to be confined to the FDG-PET positive area (7-cm-diameter range) and the rest of the tissue was tightly adherent, we decided to only replace the visible grafts. Since the FDG-PET showed no focal uptake around the aortic stentgraft, we left it undisturbed. After graft replacement with a 6-mm Fusion Bioline (Getinge, Sweden), omental flap was applied to the field and a naso-duodenal tube and percutaneously jejunal tube were placed.

The fever resolved immediately after surgery. One month postoperative FDG-PET revealed no sign of re-infection (Fig. 3E). Postoperative drainage with a naso-duodenal tube was performed to avoid any pressure accumulation on the duodenal repaired area; as the amount of drainage did not decrease, the patient remained with the tube for 3 weeks before oral intake was started. On the other hand, jejunal tube feeding was started on the third postoperative day and continued for 5 weeks. The patient was discharged after 6 weeks of intravenous triple-therapy of antibiotics (Meropenem, Daptomycin, Micafungin) and followed by 6 months of oral antibiotics (Amoxicillin/Clavulanate and Fluconazole). Discharge laboratory tests were as follows: WBC 6980/μL, CRP 0.6 mg/dL, and PCT 0.03 ng/mL. Follow-up was done every 2 months through the outpatient clinic. Six months after surgery, an abdominal CT scan showed no evidence of re-infection such as new fluid or air retention at the surgical site. During the past 1.5 years, WBC was in the 6000–7000 /μL and CRP 0.6–1.0 mg/dL range and careful progress monitoring is still ongoing.

## Discussion

A secondary aortoenteric fistula after aortic graft replacement or EVAR is a rare but devastating complication that leads to extremely high morbidity and mortality [3, 4]. Bartley et al. reported around 40% of mortality even after feasible treatment [1]. Because there are few reports of enteric fistulization complicating hybrid aortic repair with renovisceral debranching [6], there is no literature detailing the incidence and optimal treatment of this condition. Although hybrid endovascular aortic repair is considered advantageous especially for the elderly and high-risk patients, due to the avoidance of extracorporeal circulation and thoracotomy [7], surgeons should be aware of this complication and once a devastating complication happens, an optimal treatment method should be considered for patients with several comorbidities.

According to the current guideline, total excision of the infected graft is mandatory to control vascular graft infection. No definitive conclusions have yet been reached on the effectiveness of partial graft resection [5]. One case report describes the use of FDG-PET to determine the extent of the infected graft and to select partial removal [6]. We considered total removal of the graft after renovisceral debranching is more complex and potentially more invasive for an elderly man taking prednisolone, so we preserved the highly fused areas and replaced only the areas where we thought infection was spreading, which seemed to be a good choice. Areas where the grafts were exposed to purulent material were considered infected, and other areas, i.e., PET negative or showing firm adhesions, were considered uninfected.

We consider the autograft, especially saphenous vein graft, the best choice for the reconstruction, but the patient had a history of deep vein thrombosis, which precluded the use of the great saphenous vein for reconstruction. The use of autologous femoral veins has been sporadically reported [8], but their use was not considered in the present study because of uncertainty regarding their superiority over artificial grafts [9]. In addition, a rifampicin-soaked graft was not used due to a history of rifampicin allergy. In our country, cryopreserved allografts and silver-coated grafts are not commercially available. At the surgery, we could not use any of the above which are recommended in the guidelines [5]. The last choice was using GORE-TEX or Fusion Bioline for the reconstruction. This patient had a history of perigraft seroma formation maybe due to the porosity of the GORE-TEX vascular graft previously used; therefore, we determined to use Fusion Biloline due to its lower potential of perigraft seroma formation [10, 11]. Fusion Bioline seems to be superior to ePTFE graft (GORE-TEX vascular graft) in terms of patency [12], but there is no evidence regarding the use of these grafts on infection site. It is also possible that the course of the previous seroma may have been the trigger for the new infection, fistula formation, and it is important to consider what graft is feasible for initial debranching procedures.

The patient eventually recovered after partial removal of the graft, in situ graft replacement, duodenal repair, application of an omental flap, and antibiotics. These procedures provided favorable results; however, because we tried to make surgery as less invasive as possible, careful monitoring to avoid re-infection is mandatory. Additionally, it is important to consider the patient’s ability to tolerate surgery and their life expectancy when selecting patients for initial surgery.

## Data Availability

The datasets used and/or analyzed during the current study are available from the corresponding author on reasonable request.

## Abbreviations

CRP: C-reactive protein
CT: Computed tomography
EVAR: Endovascular aortic repair
FDG-PET: 18F-fluorodeoxyglucose positron emission tomography
PCT: Procalcitonin
PMR: Polymyalgia rheumatica
SUVmax: Maximum standardized uptake value
WBC: White blood cell
